# Case Report: Unilateral uterine torsion in a non-pregnant Siberian-Husky—clinical insights and implications for reproductive management

**DOI:** 10.3389/fvets.2025.1658408

**Published:** 2025-08-04

**Authors:** Ștefan Gregore Ciornei, Petru Roșca, Aurelian-Sorin Pașca, Radu Andrei Baisan, Alexandra Ciubotariu

**Affiliations:** Faculty of Veterinary Medicine, “Ion Ionescu de la Brad” Iași University of Life Sciences, Iași, Romania

**Keywords:** uterine torsion, bitch, hematometra, reproductive challenges, case report

## Abstract

Uterine torsion is a rare condition in dogs, typically associated with pregnancy or uterine pathology. A 5-year-old, female intact, Siberian-Husky presented with a history of lethargy and constipation for 3 days. Physical examination revealed pale mucous membranes and a firm, painful abdomen. Abdominal imaging revealed a thickened uterine body wall with an increased volume of mixed anechoic and heterogeneous echoic intrauterine content. Exploratory laparotomy confirmed the 360-degree torsion of the left uterine horn, and due to the extensive lesions ovariohysterectomy was deemed necessary. Postoperative clinical evaluation indicated ongoing signs of impaired oxygen delivery warranting hemotransfusion, following which progressive stabilization and complete clinical recovery was achieved. Histopathological examination showed diffuse uterine necrosis due to hypoxia from venous stasis, with inflammatory infiltrate of neutrophils, macrophages, and lymphocytes. This report contributes to the limited veterinary literature on uterine torsion in non-pregnant bitches and underscores the importance of including it in the differential diagnosis, even in young patients or when other reproductive pathology is not evident, where a lack of predisposing factors may reduce clinical suspicion. It clearly illustrates how an acute reproductive emergency can abruptly and permanently preclude any future reproductive potential from an otherwise healthy animal.

## 1 Introduction

Uterine torsion represents a rare and acute surgical emergency in the canine species, characterized by the rotation of the uterine horn along its longitudinal axis, leading to compromised vascular supply. The occurrence of this pathology is correlated with underlying conditions that cause an abnormal uterine distention, thereby explaining its increased occurrence during pregnancy. Its incidence in non-pregnant bitches although exceedingly uncommon, can rapidly result in ischemia, necrosis and systemic deterioration if left untreated (1).

A comprehensive review of databases including Web of Science and PubMed revealed that uterine torsion in non-pregnant dogs is a rarely reported and poorly characterized condition. Furthermore, a significant percent of these reports date back several years, highlighting the need for more comprehensive presentations that can emphasize the pathophysiological changes occurring in such cases (2–5). Such updates would be crucial in enhancing the understanding of uterine torsion in non-pregnant bitches, providing a more current perspective on its clinical presentation, diagnosis, and management. Notably, our case contributes valuable information by documenting a uterine torsion that occurred independently of any pre-existing morphological abnormalities of the uterus, an aspect that may refine current diagnostic considerations and broaden the clinical understanding of this condition. It enhances the understanding of a reproductive pathology that directly impairs fertility.

## 2 Case description

A 5-year-old purebred female Siberian-Husky, weighing 21 kg was presented to the Veterinary Hospital, University of Life Sciences Iași, with a history of constipation lasting 3 days. The owner noticed a minimal sanguineous discharge, initially assumed to be estrus related, although he was uncertain about the exact cause. However, during the clinical examination, we identified the normal metestrus phase. According to the owner, the female had a history of regular and normal estrus cycles, without any pregnancies. There have been no reproductive pathologies reported to date. The dog had no prior relevant medical history, and there was no confirmed evidence of foreign object ingestion.

On physical examination, the patient was alert and responsive, normothermic (38.1°C), with a heart rate of 92 bpm and respiratory rate of 24 bpm. Mucous membranes were pale, and the dog was estimated to be 5% dehydrated. The abdomen was firm and painful at palpation, with dull sound upon percussion. Superficial lymph nodes were normal.

Initial blood count revealed marked leukocytosis (WBC: 33.81 × 10^9^/L), with neutrophilia (28.82 × 10^9^/L), and moderate anemia (HGB: 6.2 g/dL; HCT: 20.9%). Platelet count was low (PLT: 108 × 10^9^/L). Biochemistry showed hyperglycemia (GLU: 150 mg/dL), hyponatremia (Na^+^: 124 mmol/L), mild hypokalemia (K^+^: 3.6 mmol/L), and hypoproteinemia (TP: 4.9 g/dL; GLOB: 2.0 g/dL). Calcium was also decreased (Ca^2+^: 8.2 mg/dL). Liver and renal parameters were within normal limits.

Radiographic examination of the abdomen revealed loss of serosal detail in the central and caudal abdomen, with severe effacement of the organs’ silhouette. A tubular-shape radiolucent area was visible the retroperitoneal space consistent with gas in the descending colon. There was a mass effect in the central abdomen displacing the small intestines cranially, suggestive for an intraabdominal mass. The ventro-dorsal view of the abdomen confirmed the mass effect, displacing the small intestine loops toward the right cranial part of the abdomen and the descending colon toward the left abdominal wall.

**Figure 1:**
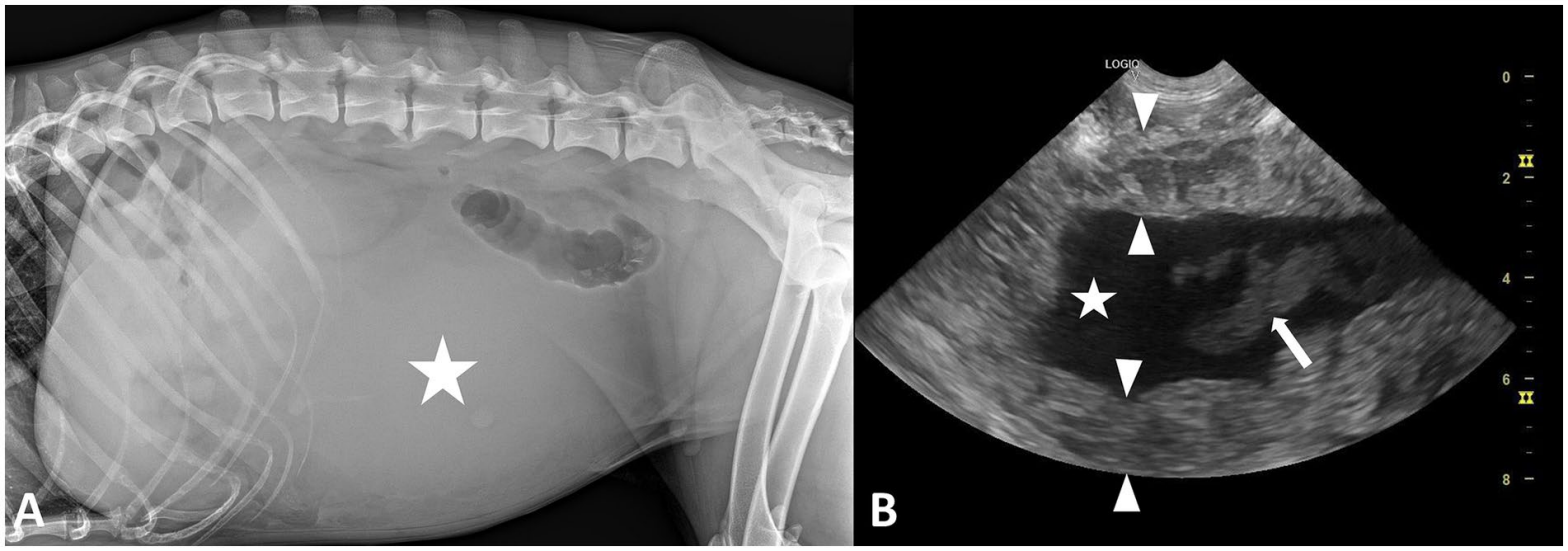
**(A)** Left lateral radiographic image of the abdomen in a 5-year-old, female intact, Siberian-Husky with uterine torsion showing loss of peritoneal serosal detail and a mass effect displacing the small intestines in the cranial abdomen; the mildly increased radiopacity in the center of the abdomen (star) is suggestive for the presence of a soft-tissue opacity mass. **(B)** Ultrasonographic image of the uterus in the same dog showing heterogeneous echogenicity and increased thickness of the uterine wall (between arrowheads) and anechoic content (star) mixed with an irregular moderately echoic content (arrow).

Abdominal ultrasonographic examination was performed with a General Electric Logiq V5 ultrasound machine equipped with a microconvex 4–10 MHz and revealed a thickened heterogeneous uterine body wall with markedly distended lumen, and a large amount of mixed anechoic and heterogeneous hypoechoic content. The left uterine horn also appeared distended with anechoic fluid. The abdominal fat in the caudal abdomen had a hyperechoic aspect. A small amount of free peritoneal fluid was also visible between the liver lobes and intestinal loops. These features were suggestive of a uterine pathological process and an exploratory laparotomy was recommended (Figure 1).

The patient was premedicated with buprenorphine 0.02 mg/kg IM (IM) and anesthesia induction was made with ketamine 100 mg/mL and diazepam 5 mg/mL administered as a 1:1 ratio with an anticipated total dose of 1 mL/20 kg IV. It was intubated and anesthesia maintained using isoflurane as an inhalant anesthetic.

**Figure 2:**
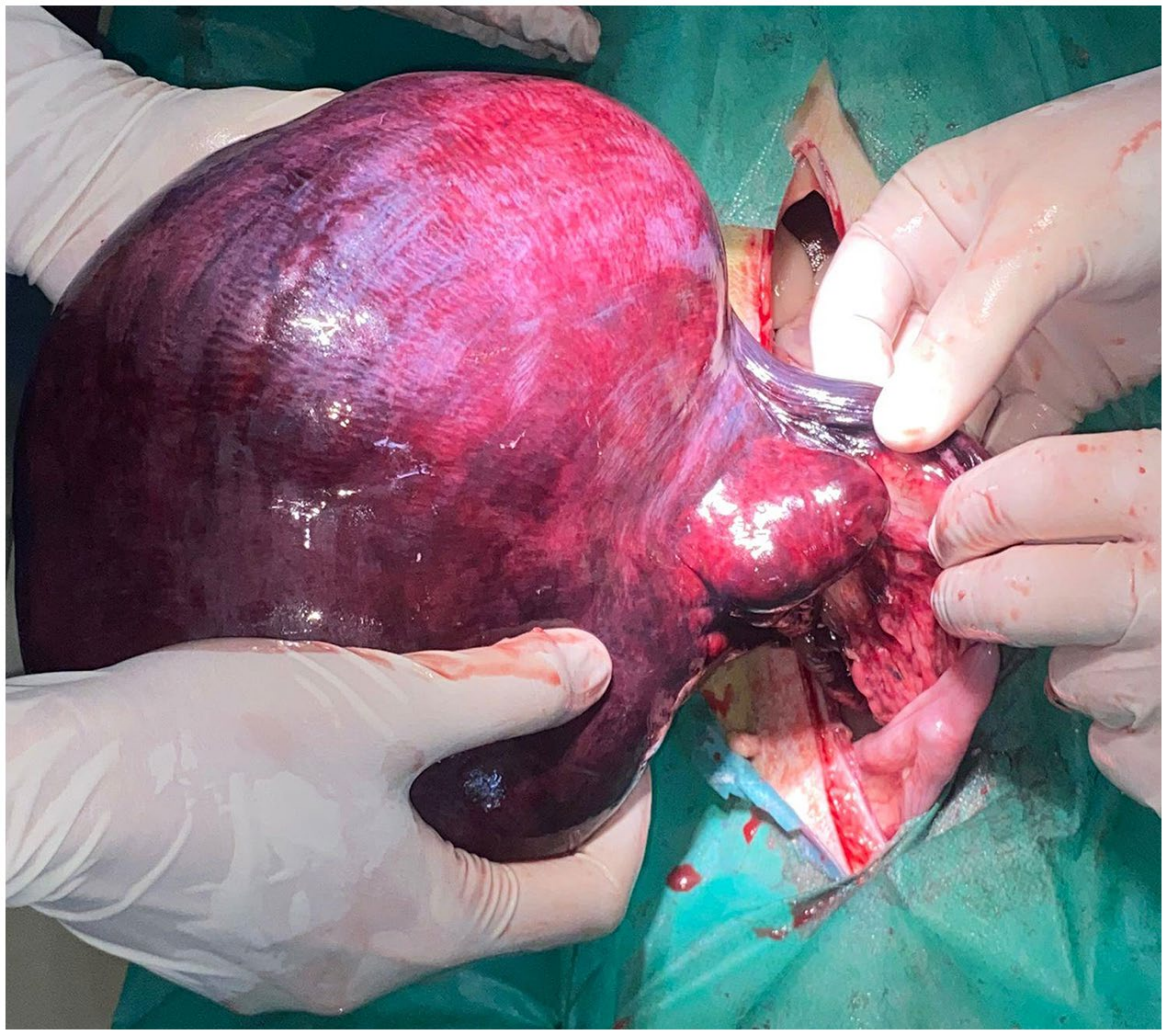
Intraoperative aspect of the twisted uterine horn prior resection. Significant distention and vascular congestion can be observed.

The female was placed in dorsal recumbency and the surgical area clipped from xiphoid to pubis. A post-umbilical ventral midline incision was performed. Exploratory laparotomy revealed that the structure initially appearing as a large reniform mass in the mid-portion of the left uterine horn was, in fact, the uterus itself, which had undergone a 360-degree torsion along its longitudinal axis (Figure 2). It had a purple-brown appearance and was elastic at palpation. No cystic or neoplastic lesions were observed on ovaries. The examination of the remaining abdominal viscera did not reveal any abnormalities.

Although not initially desired, ovariohysterectomy became necessary due to the extent and the irreversible nature of the lesions. Unilateral ovariohysterectomy and oocyte retrieval were recommended as alternative options; however, the owner declined both, opting instead for definitive surgical resolution. The ovarian pedicles were clamped and ligated using 2/0 synthetic monofilament absorbable sutures, followed by transfixation ligation of the uterine body with 2/0 PDS. Laparorraphy was performed by closing the muscular layer using 0 PDS in a simple continuous pattern, followed by a subcutaneous closure with 2/0 PDS in a similar fashion. The skin was closed using interrupted sutures.

Postoperative analgesia was provided with 0.02 mg/kg buprenorphine administered IM every 8 h.

After surgery the patient showed persistent clinical signs of inadequate oxygen delivery, including pale mucous membranes, sustained tachycardia, and marked lethargy. Considering the hematocrit (20.9%) and hemoglobin concentration (6.2 g/dL), which were close to, or already below the transfusion thresholds recommended (PCV < 20% or Hb < 7 g/dL in acute anemia) (6), hemotransfusion was considered necessary. The patient was hospitalized for 3 days and subsequently released in good clinical condition.

At the 2-week follow-up, no postoperative complications were seen. At 1-month follow-up the dog was in good general condition, having a normal appetite and typical activity levels.

**Figure 3:**
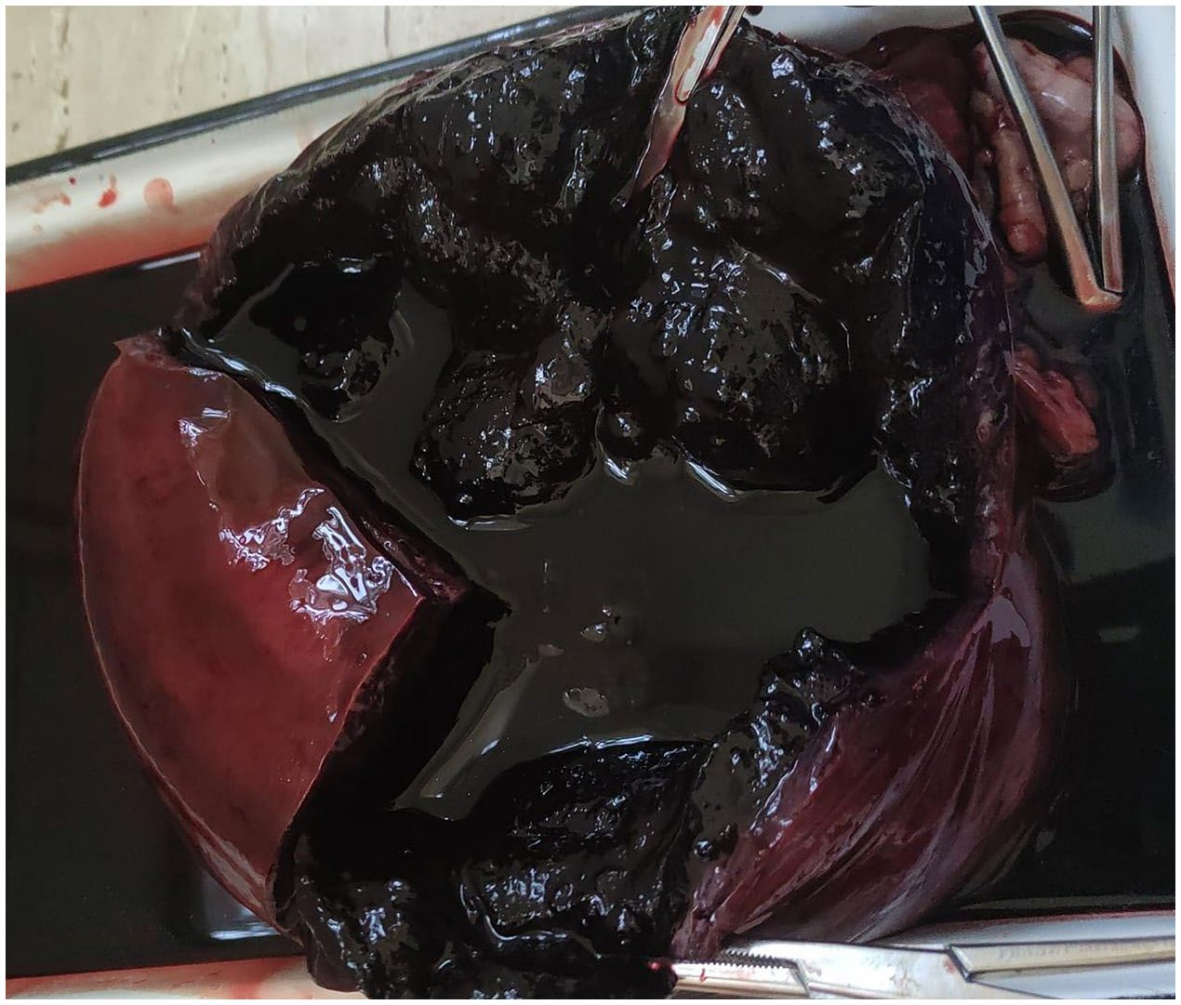
Gross appearance of the sectioned uterine horn. Note the luminal dark-red fluid and clotted blood consistent with advanced hemometra.

Macroscopic examination of the excised uterus revealed marked distension of the uterine lumen, filled with dark sanguinous fluid and large amount of coagulated blood, consistent with hematometra (Figure 3). It measured 28 × 16 cm and weighed 3.2 kg.

Fragments of the uterine wall were sampled for histopathologic examination. The pieces were immersed in 10% buffered formaldehyde for 48 h, followed by paraffin-embedding processing. Paraffin embedding was accomplished with a Leica TP1020 rotary tissue processor (Leica Microsystems GmbH, Germany), and SLEE CUT 6062 microtome (SLEE Medical GmbH, Germany) was used for sectioning at 5-μm thickness. After deparaffinization, sections were stained by the Masson trichrome method. Qualitative histologic examination was performed using a Leica DM microscope (Leica Microsystems GmbH, Germany) with Leica ICC50 HD, 5 mpx histologic camera (Leica Microsystems GmbH, Germany). Photomicrographs were taken using Leica Application Suit Software (LAS) version 4.2. The entire protocol was performed under standard conditions for all tissue samples.

**Figure 4:**
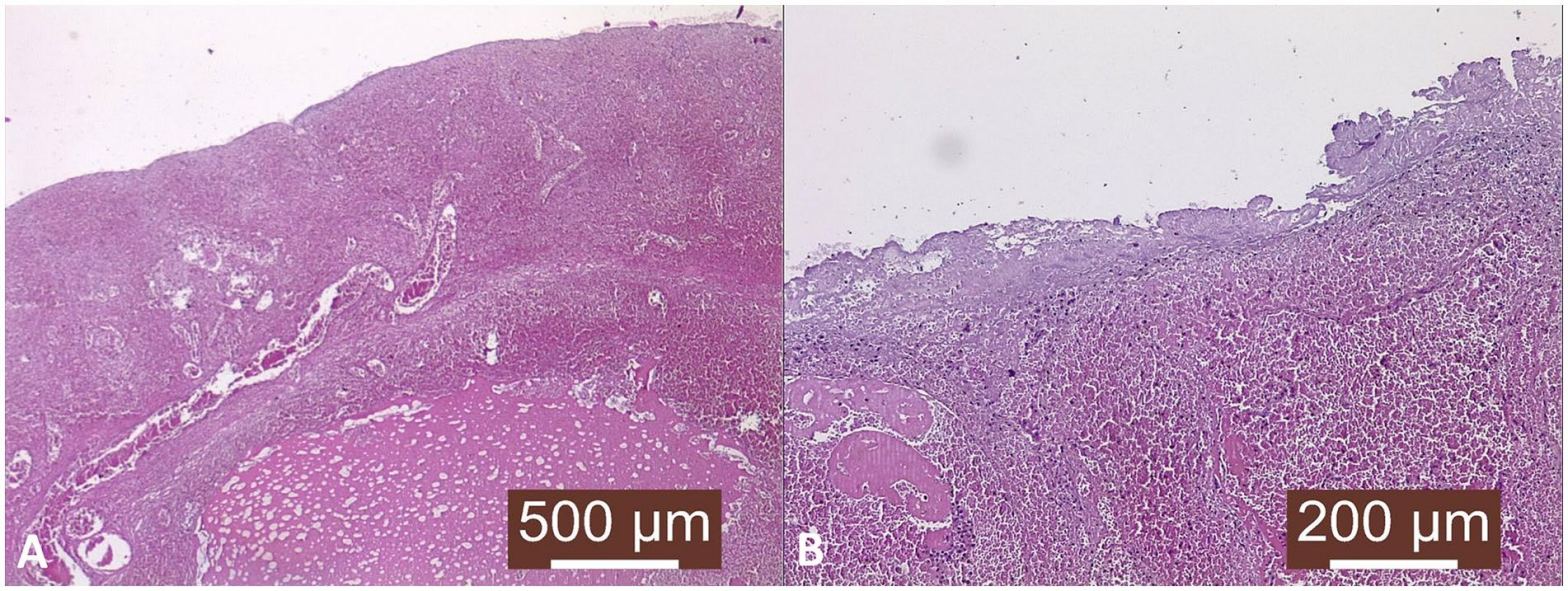
Histopathological examination. **(A)** Uterine wall—venous stasis and venous infarction. Profuse transudate dilacerating uterine structures, especially myometrium; Masson’s trichrome stain, ×40. **(B)** Severe necrosis of the endometrium and underlying structures, inflammatory reaction in the submucosa, vascular distention (stasis); Masson’s trichrome stain, ×100.

Histological examination revealed diffuse necrosis of the uterine parietal structures: mucosa and glandular components, along with leukocyte infiltration due to deep hypoxia associated with local venous stasis. Inflammatory transudate and neutrophilic leukocytes, macrophages, and lymphocytes were also present within uterine structures (Figure 4).

## 3 Discussion

Reproductive pathology in female dogs rarely includes uterine torsion (7), a condition more frequently reported in cattle and primarily attributed to excessive fetal movement, uterine asymmetry and ruminal pressure (8). In bitch is most frequently associated with late pregnancy, or rarely with hematometra, pyometra, and focal uterine adenomyosis.

The uterus is a suspended organ, supported by the ovarian pedicle and broad ligament, making it susceptible to torsion under certain pathological conditions. Furthermore, following torsion, compromised venous and lymphatic drainage may further aggravate the distension due to circulatory disruption, leading to a progressive cycle of congestion and further enlargement. The presenting symptoms of our patient are consistent with those described in the literature on uterine torsion, which commonly include lethargy, inappetence and vaginal discharge. Depending on the degree of torsion and uterine enlargement, abdominal distention may also be observed (9, 10).

Unlike other cases described in the literature where uterine torsion is typically associated with predisposing structural abnormalities such as tumoral growth or cystic endometrial hyperplasia, the present case lacked any of these factors. The torsion observed most likely resulted from severe vascular compromise. These changes led to hematometra, progressive distension, and degenerative alterations of the uterine wall, ultimately resulting in mechanical imbalance and axial rotation of the uterine horn. Hematometra may act both as an etiologic trigger for uterine torsion and as a secondary manifestation from it. This pathology is also linked to intrauterine bleeding following parturition due to local injuries and subinvolution of placental sites, anticoagulant rodenticide intoxication, coagulation deficiency, neoplasia, excess effusion from hyperplastic endometrium after long-term suppression with progesterone (10–13).

Advanced age has been implicated in several cases as a contributing factor in uterine torsion, promoting relaxation and weakening of the uterine ligaments, thereby increasing the mobility of the uterus and predisposing it to abnormal rotation (2, 14). However, as this patient was only 5 years old, less than half the age reported in the limited number of documented cases, the occurrence of this pathology highlights the unusual nature of the case and suggests the involvement of additional predisposing factors. In non-pregnant female dogs, uterine torsion is typically associated with predisposing factors such as uterine masses (polyps or hemangiomas), ligament laxity in multiparous bitches, or sudden inertial forces from movements like falling or rolling (9). These factors often act in combination. However, in the present case, torsion occurred in the absence of any discernible structural abnormality, suggesting an atypical etiology and making it clinically noteworthy.

A key strength of this case report lies in the detailed clinical and surgical documentation, which contributes to the limited literature on this rare condition and may assist clinicians in timely recognition and decision-making. It further illustrates the potential consequences of delayed intervention and the necessity of prompt surgical management. However, limitations include the lack of generalizability due to the single-subject nature of case reports. Despite these limitations, the case underscores a critical and often overlooked condition in small animal reproductive medicine.

Although uterine torsion is rare in non-pregnant dogs, its consequences are often severe and irreversible (14). The associated vascular compromise commonly results in ischemic necrosis of the uterine tissue, leading to a substantial reduction of the fertility potential, unilateral hysterectomy (15) or even ovariohysterectomy with major implications for the application of reproductive biotechnologies. Once the uterus is damaged beyond function, procedures such as artificial insemination (AI), embryo transfer (ET), or uterine flushing for embryo become impossible (16). Even oocyte retrieval may be compromised if ovarian blood flow is affected (17–20). In breeding animals with genetic value, or in programs focused on preserving rare breeds, this loss represents more than a clinical outcome, it is a lost opportunity for genetic contribution. This case clearly illustrates how an acute reproductive emergency can abruptly and permanently exclude an otherwise healthy female from any future use in assisted or natural breeding. It emphasizes the need for earlier and more routine reproductive assessment in breeding bitches, even when clinical signs are absent, to avoid irreversible losses of reproductive potential.

## 4 Conclusion

This case provides further evidence on uterine torsion in dogs, particularly in the absence of structural abnormalities. These findings suggest that, even in cases lacking classical predisposing lesions, uterine torsion represents a distinct clinical entity with a good prognosis when managed surgically in a timely manner, preventing a potentially fatal outcome. Uterine torsion should be routinely considered as a differential diagnosis in intact bitches presenting with vulvar discharge, as it can compromise fertility and overall reproductive health, making prompt recognition and timely intervention essential.

## Data Availability

The original contributions presented in the study are included in the article/supplementary material, further inquiries can be directed to the corresponding authors.
